# Relationship between Nutrition, Lifestyle, and Neurodegenerative Disease: Lessons from *ADH1B*, *CYP1A2* and *MTHFR*

**DOI:** 10.3390/genes13081498

**Published:** 2022-08-22

**Authors:** Shila Barati, Carlo Fabrizio, Claudia Strafella, Raffaella Cascella, Valerio Caputo, Domenica Megalizzi, Cristina Peconi, Julia Mela, Luca Colantoni, Carlo Caltagirone, Andrea Termine, Emiliano Giardina

**Affiliations:** 1 Genomic Medicine Laboratory-UILDM, Santa Lucia Foundation IRCCS, 00179 Rome, Italy; 2 Data Science Unit, Santa Lucia Foundation IRCCS, 00143 Rome, Italy; 3 Department of Biomedicine and Prevention, Tor Vergata University, 00133 Rome, Italy; 4 Department of Biomedical Sciences, Catholic University Our Lady of Good Counsel, 1000 Tirana, Albania; 5 Department of Clinical and Behavioral Neurology, Santa Lucia Foundation IRCCS, 00179 Rome, Italy

**Keywords:** nutrition, neurodegeneration, epigenetics, nutrigenomics, SNPs, physical exercise, microbiota, metabolism, Alzheimer’s disease, Parkinson’s disease

## Abstract

In the present review, the main features involved in the susceptibility and progression of neurodegenerative disorders (NDDs) have been discussed, with the purpose of highlighting their potential application for promoting the management and treatment of patients with NDDs. In particular, the impact of genetic and epigenetic factors, nutrients, and lifestyle will be presented, with particular emphasis on Alzheimer’s disease (AD) and Parkinson’s disease (PD). Metabolism, dietary habits, physical exercise and microbiota are part of a complex network that is crucial for brain function and preservation. This complex equilibrium can be disrupted by genetic, epigenetic, and environmental factors causing perturbations in central nervous system homeostasis, contributing thereby to neuroinflammation and neurodegeneration. Diet and physical activity can directly act on epigenetic modifications, which, in turn, alter the expression of specific genes involved in NDDs onset and progression. On this subject, the introduction of nutrigenomics shed light on the main molecular players involved in the modulation of health and disease status. In particular, the review presents data concerning the impact of *ADH1B*, *CYP1A2,* and *MTHFR* on the susceptibility and progression of NDDs (especially AD and PD) and how they may be exploited for developing precision medicine strategies for the disease treatment and management.

## 1. Introduction

Most neurodegenerative diseases (NDDs) have been associated with genetic and environmental factors, with regard their potential to influence disease susceptibility and progression [1]. Concerning genetic factors, the study of the human genetic variability allowed identifying different variants (mainly single-nucleotide polymorphisms, SNPs) associated with an increased susceptibility to develop NDDs among worldwide populations. In addition, external factors (age, environment/lifestyle, diet, and comorbidities) have also been shown to crosstalk with the genome, by means of epigenetic modifications, which essentially modulate gene expression in different tissues without altering the DNA sequence. Actually, the alteration of the epigenome has been described as a contributing factor in the development and progression of NDDs [1].

Concerning environmental factors, smoking habit is a key risk factor for the development of NDDs as well as the chronic exposure to contaminants such as heavy metals and pesticides [2,3,4]. In addition, there is a close relationship between dietary habits and NDDs [5], since a high consumption of saturated fatty acids exacerbate neurodegeneration by increasing lipid peroxidation and, in turn, oxidative stress [6]. Moreover, the uncontrolled intake of saturated fatty acids has been assumed to trigger the inflammatory response, recruit the peripheral immune cells into the central nervous system (CNS) and, thus, contribute to the exacerbation of disease symptoms [7]. Furthermore, studies revealed that the consumption of long-chain fatty acids (LCFAs) have been found to worsen disease symptoms, whereas the intake of short-chain fatty acids (SCFAs) have been shown to improve the disease course [8].

Minerals and vitamins exert multiple effects on neuronal signalling and communication. In fact, B vitamins affect fiber myelination and neuronal survival, whereas E vitamins support mitochondrial function, acting as an effective antioxidant factor [9].

In this scenario, the different nutritional plans have been shown to affect the susceptibility and progression of NDDs, such as Alzheimer’s disorder (AD), Parkinson’s disease (PD), and multiple sclerosis (MS).

On this subject, the therapeutic potential of the ketogenic diet has been studied in such conditions [5]. In particular, the ketogenic diet consists of a low-carbohydrate, adequate-protein, and high-fat intake, raising fatty acid oxidation to ketone bodies and, thereby, replacing glucose as the primary energy source [10,11,12]. Therefore, this diet has been shown to enhance neuronal bioenergetics by promoting mitochondrial biogenesis, stabilizing synaptic function, triggering the production of brain-derived neurotrophic factor (BDNF) and, thereby, promoting the neuroprotective functions of the brain [13].

Moreover, a protective role for the Mediterranean diet has been described in relation to different multifactorial diseases (including stroke, age-related macular degeneration, and NDDs) [14]. The Mediterranean diet is based on a high consumption of monounsaturated and polyunsaturated fatty acids and antioxidants (included in fruit, vegetables, fish, and olive oil). Interestingly, this kind of diet has been reported to have anti-depressive effects and improve cognitive ability, thus, reducing the susceptibility to AD and PD [15,16,17].

In addition, caloric restriction has also been demonstrated to protect against neurodegeneration. In fact, this has been shown to activate a mild chronic stress response in neurons, promoting, in turn, an increased production of neurotrophic factors (such as BDNF and chaperones), which prevent protein aggregation and neuronal death [18].

Importantly, a recent study found an association between the Mediterranean-DASH Intervention for Neurodegenerative Delay (MIND) diet and a decreased risk of dementia. In particular, the MIND diet contains recommendations regarding several foods (i.e., green leafy vegetables, whole grains, other vegetables, nuts, berries, beans, fish, poultry, olive oil, and wine) considered to be healthy for the brain, in contrast to others classified as unhealthy (red meat, butter, cheese, pastries, fast fried food, and sweets) [19]. Hence, healthy food components (i.e., polyunsaturated fatty acids, antioxidants including resveratrol, blueberry polyphenols, curcumin, sulphoraphane, and salvionic acid) as well as caloric restriction and physical activity may counteract ageing and any associated neurodegenerative diseases [20].

Over dietary habits and nutritional components, gut microbiota has been shown to exert multiple functions (including the resistance to pathogens and maturation of the immune system) and to participate in brain physiology as well as neurodegenerative conditions [2]. In particular, the diversity and composition of gut bacteria regulates the amount of microbiota-derived metabolites, neurotransmitters, and the SCFAs, which constitute the major end-products of microbial fermentation in the gut [21]. Indeed, brain function, behavior, and cognition can also be modulated by gut-derived signaling molecules, which can communicate with the brain by neural communication, endocrine signaling, and the immune system [5,22,23]. The disruption of the microbiome composition (i.e., dysbiosis) can be detrimental to human health and immunity, resulting thereby, in a higher susceptibility to disease conditions, including NDDs (AD, PD) [24,25,26,27]. In addition, the disease severity of NDDs can also be affected by microbiome metabolites by two mechanisms: (i) immune-mediated neurodegeneration, and (ii) the direct effects of microbiome-derived metabolites on CNS cells.

Given these premises, the present review aims to provide an overview of the main features involved in the susceptibility and progression of NDDs. In particular, the impact of genetic and epigenetic factors, nutrients, and lifestyle will be discussed with the purpose of highlighting their potential application for promoting the management and treatment of patients with NDDs.

## 2. Diet and NDDs: Focus on AD and PD

### 2.1. AD

AD is a complex disorder characterized by an irreversible and progressive decline of cognitive functions, resulting in memory loss, an impairment in decision-making, and an inability to carry out basic daily activities [28,29]. These clinical signs affect the lifestyle of patients and their families causing physical, psychological, social, and economic changes [28]. People suffering from AD are estimated to account for 35.6 million and 7.7 million new cases are diagnosed every year [30]. Moreover, the number of people with AD will dramatically triple over the next 40 years with a notable increase in costs for diagnosis and treatment [31]. The main pathological hallmarks of the AD brain include neuron loss at the level of the hippocampus and neocortex/entorhinal cortex, and atrophy of the temporal and parietal cortex [32]. Additional neuropathological features of the disease are the presence of extracellular β-amyloid (Aβ) plaques and intracellular neurofibrillary tangles (NFTs), which are caused by the alteration of amyloid precursor protein (APP) and the increasing of phosphorylated-tau (P-Tau) protein, respectively [33].

The main genetic risk associated with AD is represented by the *epsilon 4* variant of the *Apolipoprotein E* (*APOE*, 19q13.32) gene. This encodes a protein mainly involved in lipid transport. In fact, APOE is the primary component of plasma lipoproteins, and it is responsible for their production, conversion, and clearance. Although it associates with very low-density lipoproteins (VLDL) and intermediate density lipoproteins (IDL), APOE preferentially binds to high-density lipoproteins (HDL). It is crucially involved in the metabolism of plasma and tissue lipid metabolism, especially regarding cholesterol homeostasis. Importantly, APOE is also implicated in lipid transport in the CNS as well as in the regulation of neuron survival and sprouting [33,34,35]. In particular, this role provided a link for aberrant lipid metabolism and the onset of AD [36,37]. In fact, higher levels of LDL and lower levels of HDL were shown to be associated with increased deposition of Aβ in the brain [36].

Studies on AD mice carrying the *APOE4* allele revealed a strong negative impact after diet-induced weight gain, as shown by the higher incidence of metabolic disturbances such as obesity, hypercholesterolemia, and type 2 diabetes, compared to AD mice carrying the *APOE3* allele [38,39,40].

In addition, insulin and insulin-like growth factor-1 (IGF-1) are known to participate in neuronal development and survival through the stimulation of synaptic plasticity and long-term potentiation, contributing thereby to learning and memory functions. In particular, insulin modulates tau protein’s phosphorylation, and, thus, has been involved in AD etiopathogenesis [41].

Moreover, studies on animal models showed that caloric restriction has a protective effect against AD onset and progression, through the decreasing of Aβ deposition and the enhancement of neurogenesis [42,43].

A research study has shown that the intake of daily food supplemented with ascorbic acid for eight weeks resulted in a reduction of advanced glycation end-products [44]. In addition, this approach was also associated with enhanced plasma HDL levels and LDL composition, exerting thereby protective effects against systemic inflammation and atherosclerosis. Furthermore, the study showed an inverse correlation between ascorbic acid and the expression of several miRNAs (such as miR155 levels), suggesting that high doses of ascorbic acid may decrease inflammation via the modulation of miRNA levels [44]. Given that miR155 and inflammation are key players involved in AD etiopathogenesis and progression, their possible modulation by ascorbic acid supplementation represent a possible treatment strategy for AD. Moreover, a protective effect against AD onset has been shown for the concomitant supplementation of ascorbic acid and vitamin E [45].

Observational human studies revealed that AD patients show an altered microbiome composition [46,47,48,49,50]. Supporting this evidence, a study on mice described a strong association between murine microbial composition and the *APOE* genotype, which was independent from disease status and sex [51,52]. Hence, AD onset and progression could be affected by microbial composition, which, in turn, may be affected with specific AD-associated genetic factors, such as the *APOE* genotype [2].

### 2.2. PD

PD is a complex movement disorder characterized by high heterogeneity in terms of clinical presentation and environmental and genomic contributing factors [1]. It is caused by the progressive loss of dopaminergic neurons in the *Substantia Nigra pars Compacta* (SNc) and the formation of abnormal aggregates of protein referred to as Lewy bodies. PD affects more than 10 million people worldwide, although the prevalence can differ because of age, male sex, and geographic position. This disease has dramatic effects on a patient’s life as well as on the overall health system. Clinical features associated with PD include a resting tremor, rigidity, bradykinesia, and postural instability, although patients can also experience non-motor symptoms (mainly neuropsychiatric, olfactory, and sleep disturbances) [1].

In the last decades, dietary habits, microbiota and metabolism have had a strong impact on PD incidence, revealing a great contribution in dopaminergic neurotoxicity [2]. The relationship between diet and PD shows an association with specific foods. In particular, milk intake is the most reliable dietary factor associated with an increased susceptibility to PD, even if it is not explained by vitamin D levels, calcium, or the amount of dairy fats [53,54]. Instead, many studies have revealed that coffee consumption is associated with a lower risk of PD. In fact, it appears to benefit males more than females, and, accordingly, sex hormones may be important modifiers of caffeine’s protective function [55,56,57]. Caffeine blocks the adenosine A2a receptor acting as an antagonist; this blockage may inhibit glutamate excitotoxicity, increasing neuronal survival. Data also revealed that caffeine downregulates inflammatory cytokines and microglia activation, exerting its neuroprotective role [58,59].

Coenzyme Q10 and fish oil among nutritional supplements reduce the rates of PD progression, having a statistically significant correlation with PD [20].

Considering that some symptoms observed in the early phases of PD occur at the gastrointestinal level, the dysbiosis may play a triggering role in the disease etiopathogenesis, suggesting that it may represent a potential treatment target for PD [22,60]. In fact, patients suffering with PD displayed a lower prevalence of protective and anti-inflammatory bacterial species, in combination with higher amounts of proinflammatory species, which were associated with postural instability, worse motor function, and fluctuations. Of note, aggregates of α-synuclein have been detected in the sigmoid mucosa of patients 2–5 years before the onset of disease symptomatology. Thus, it has been hypothesized that the α-synuclein is subsequently moved to the brain by means of a prion-like mechanism or inflammatory and oxidative stress processes. In addition, variants of the genes coding for peptidoglycan recognition proteins have also been associated with PD risk, suggesting that they may influence the microbiota composition and the immune response toward commensal and harmful bacteria. Consequently, gut mucosa would be more vulnerable and prone to inflammation, contributing thereby to the accumulation of α-synuclein and to the initiation of the neuropathological cascade responsible for PD [1].

## 3. Genetic and Epigenetic Factors Involved in Nutrients Metabolism and NDDs

### 3.1. Genetic Factors

Recent studies focused their attention on the genetic and epigenetic features that influence the ability of nutrients to modulate health status, giving rise to the new line of research known as nutrigenomics. On this subject, various protective pathways such as caloric restriction and lipid and vitamins metabolism have been connected to age-related disease, including NDDs. This approach opens the way for introducing new action plans for promoting neuroprotection exploiting diet and finding new natural substances that can be more effective. Indeed, the identification of such nutrigenomic factors should be able to induce health-promoting genes and reduce the expression of disease-promoting genes and other yet unknown pathways.

To this purpose, the study of genetic variability highlighted the association of 11 SNPs with significant effects on nutrition and health. In particular, the rs762551 (C/A) in the *CYP1A2* (15q24) gene is involved in caffeine metabolism, whereas the rs1229984 (T/C) and rs2066702 (G/A) in the *ADH1B* (4q23) gene are associated with alcohol metabolism. Moreover, the rs738409 (C/G) in the *PNPLA3* (22q13.31) gene plays a role in increasing fat accumulation and, interestingly, it is involved in non-alcoholic fatty liver disease. The rs9939609 (T/A) is localized in the *FTO* (16q12.2) gene and it is involved in obesity, appetite, and increasing adiposity, whereas the rs174537 (G/T) in the *FADS1* (11q12.2) gene is involved in long-chain fatty acid biosynthesis. Among the other SNPs, a high emphasis has to be given to rs7412 (C/T) and rs429358 (T/C), which are located in the *APOE* gene that represents the main genetic risk factor for AD and it is critical for lipid metabolism. Finally, the rs1801133 (G/A) in the *MTHFR* (1p36.22) gene is involved in folate metabolism, whereas the rs7041 (A/C) and rs4588 (G/T) in the *GC* (4q13.3) gene affects the transport of vitamin D [60].

**Figure 1:**
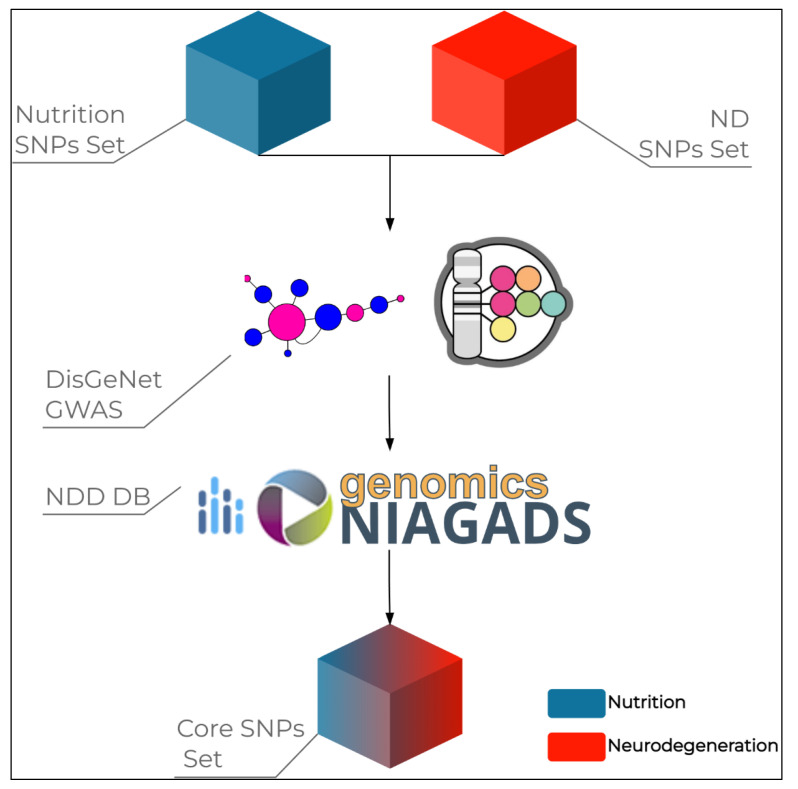
The research workflow utilized to identify SNPs with effects both on nutrition and neurodegenerative diseases.

Taking into account the above-mentioned nutrigenomic SNPs, we investigated the hypothesis that 11 SNPs known for their effects on nutrition and health were also associated with NDDs. Therefore, we performed research among database and literature studies searching for data supporting this thesis (Figure 1).

Firstly, we utilized the DisGeNET platform (v7.0, https://www.disgenet.org/home/, accessed on 11 August 2022), which allows the research of genes and variants associated with human disease thanks to the integration data collected from expert curated repositories (including GWAS catalogues, animal models, and scientific literature). To this purpose, we queried our SNPs of interest into the platform and we filtered the results to see those related to the “Nervous System Diseases” class. Subsequently, we selected the SNPs associated with NDDs, especially AD and PD. We then performed a search on public databases (namely, GWAS catalogue, AlzGene, PdGene, NIAGADS Alzheimer’s Genomics Database) and literature, searching for data concerning the investigation of the selected SNPs in the context of AD, PD, or other factors that may influence their susceptibility, progression, or treatment response. To this purpose, we utilized the rs number and the name of the disease (namely, AD and PD) as keywords. As a result, we were able to prioritize four SNPs, that are rs7412 (*APOE*), rs429358 (*APOE*), rs1229984 (*ADH1B*), rs762551 (*CYP1A2*), and rs1801133 (*MTHFR*). As expected, SNPs of *APOE* were found to be associated with the risk of AD and PD. In addition, the *ADH1B* and *MTHFR* variants were associated with both AD and PD, whereas the SNP of *CYP2A1* was linked only to PD risk. Concerning the four SNPs of interest and the disease-related factors, cognitive impairment (SNPs of *APOE*, *ADH1B*, *MTHFR*), alcohol abuse (*ADH1B* variant), caffeine consumption (SNPs of *CYP2A1*, *MTHFR*), and homocysteine levels (*MTHFR* variant) appeared as the mostly represented traits. Indeed, this result reflects the biological functions of these genes in human health and disease and point to *ADH1B*, *CYP1A2,* and *MTHFR* as candidate nutrigenomic factors able to influence the susceptibility and progression to NDDs [61,62,63]. Moreover, these genes and their related variants deserve further investigation because of their possible application to optimize and personalize the treatment to such conditions.

### 3.2. Epigenetic Factors

Over genetic variability, the epigenetic elements have also been studied as potential nutrigenomic factors. In particular, epigenetic elements are able to shape and modulate gene expression in response to external stimuli (age, environment/lifestyle, diet, and disease condition). To date, at least three epigenetic mechanisms are known to impact gene expression, namely DNA methylation, histone modification, and non-coding-RNAs [64].

Diet directly influences the gene expression profile of cells during a lifetime [2]. In particular, high levels of SCFAs are associated with lower histones acetylation that, consequently, can result in chromatin remodelling and, in turn, in the modification of gene expression [65].

Studies on human subjects indicated that epigenetic modifications could also be influenced by physical activity [66]. In fact, some studies have demonstrated that physical activity can modulate histone acetylation in different tissues, triggering chromatin changes, leading thereby to the transcription or repression of specific genes associated with multifactorial disorders (such as cancer and NDDs) [67,68]. In the last few years, various studies have shown that physical activity induces epigenetic changes that affect the CNS plasticity. Among the genes mostly affected by epigenetic regulation, it is important to mention *synuclein α* (*SNCA*, 4q22.1); *parkin RBR E3 ubiquitin protein ligase* (*PARK2*, 6q26); *Parkinson disease 16* (*PARK16*, 1q32) and *leucine-rich repeat kinase 2* (*LRRK2*, 12q12); [69,70,71,72,73,74,75].

Concerning ncRNAs, miRNAs have been extensively investigated because of their critical role as post-transcriptional regulators and for their expression in both physiological and pathological conditions. In addition, miRNAs can be detected in several body fluids, such as blood, saliva, urine, and cerebrospinal fluid. In the last decades, several research efforts have been made to disclose the role of miRNAs in neurogenesis and neurodegeneration [64]. Actually, miRNAs are known to be involved in the production and degradation of toxic proteins accumulating in the brain as well as in neuronal death. In addition, they can be expressed as a result of cellular or tissue damage, aging, and dysfunction of pro-survival proteins. Currently, miRNA-29a, miRNA-29b, miRNA-34a, miRNA-103, miRNA-107, miRNA-125a, miRNA-146a, miR-155, miR-196a, miR-499a, and many others have been involved in neuroinflammation, mitochondrial dysfunction, synaptic transmission, endosomal–lysosomal dysfunction, apoptosis, oxidative stress, and membrane and intracellular trafficking [64].

## 4. Physical Activity and NDDs

The onset and progression of NDDs are also influenced by physical exercise, diet, and stress [5]. Interestingly, physical exercise could influence Aβ levels and slow AD progression [76]. Preclinical studies demonstrated cognitive improvements in PD patients, promoted by physical exercise [77,78]. The reason for the improvement of disease symptoms may be related to the increase in neurotrophic factors (BDNF, NGF, VEGF) levels, highlighting physical exercise as a neuroplasticity promoter [79]. Preclinical studies revealed that physical activity is able to increase antioxidant enzymes, anti-inflammatory cytokines, and anti-apoptotic proteins in intestinal lymphocytes, leading to an overall reduction in gut inflammation [80,81]. Moderate physical activity maintains the intestinal blood flow, modulating the gastrointestinal motility, and reducing inflammation [82]. Instead, excessive physical exercise can produce a stress response that result in the increasing of cortisol and epinephrine levels [83] and a reduction in blood supply to the intestinal epithelium, which, in turn, leads to damage of the gut barrier, inflammation, and gastrointestinal distress [82].

Physical exercise has been described as a protective factor against cognitive decline and AD. The effects of physical exercise can be modulated by genetic factors, as suggested in AD patients, where *APOE4* allele carriers were more responsive to the beneficial effects of physical activity compared to non-carriers [84].

The level of physical exercise has been shown to influence the methylation profile of the *MTHFR* gene and, consequently, the levels of homocysteine, particularly among the elderly [85]. In addition, polymorphisms of *MTHFR* that are known to affect protein activity and homocysteine levels have also been associated with differential physical performance [86,87,88]. Interestingly, physical activity has been correlated with lower levels of homocysteine, and thus, it could be combined with nutritional interventions to modify the risk for chronic disorders and their complications [85,89,90]. In this perspective, the evaluation of the genetic (i.e., genetic variants) and epigenetic biomarkers (i.e., methylation profile) of *MTHFR* activity could be helpful for developing strategies for preventing or monitoring disease course and its-related complications. Concerning the relationship between CYP1A2 and physical activity, most of the studies investigated the interaction between caffeine intake and *CYP1A2* genotype in relation to the differential response to exercise performance and the regulation of blood pressure [91,92,93,94]. Indeed, these studies investigated the utility of caffeine supplementation on the basis of a *CYP1A2* genotype as a possible nutrigenomic approach addressed to improve the exercise performance of athletes for many sports. However, caution should be used before translating such findings to non-athletic and older individuals, for which further studies should be performed [92].

The inflammatory process plays an important role in the pathogenesis of neurodegeneration and an improvement of the inflammatory profile in response to specific training programs has been suggested. Several studies have reported that interleukin-12 (IL-12)-associated genes, such as interleukin 12A (*IL12A, 3q25.33)*, interleukin 12B (*IL12B, 5q33.3*), interleukin 12 receptor subunit β 1 (*IL12RB1, 19p13.11*), and interleukin 12 receptor subunit β 2 (*IL12RB2, 1p31.3*), could be associated with cognitive aging through gene–physical activity interactions [95]. IL-12 is known to participate in neuroinflammatory processes and it has been associated with AD and mild cognitive impairment [95,96].

On this subject, it has been shown that aged people with reduced physical activity showed a higher cognitive decline and higher levels of IL-12β compared to active elderly people, therefore supporting the role of physical activity on individual inflammatory profiles [97]. Associations between IL-12-associated genes and late-onset AD have been reported for rs116910715, rs78902931, and rs78569420 in *IL12A*; and rs730691 in *IL12B* and rs3790558, rs4655538, rs75699623, rs1874396 in *IL12RB2*. A reduction of circulating levels of pro-inflammatory cytokines has been also suggested as a consequence of physical activity, supporting the role of exercise as an epigenetic modulator [95].

Additionally, physical activity-related switch genes involved in cognition and neurodegeneration have been identified [98]. These genes act via the upregulation of synaptic signaling pathways, conferring neuroprotection in AD and PD, and via the downregulation of genes involved in inflammation in AD, hence playing an important role in disease pathogenesis [98].

## 5. Discussion and Conclusions

The present review discussed the genetic, epigenetic, nutrigenomic, and lifestyle factors that could influence the susceptibility and progression of NDDs, with a special focus on AD and PD. In this context, many protective and risk genetic variants known to affect lifestyle have been also associated with the onset and progression of one or more NDDs. Moreover, epigenetic modifications have been shown to contribute to such conditions and thereby represent possible targets to develop treatment strategies to be combined with nutritional supplements. On this subject, the identification of nutrigenomic factors represent a promising opportunity to promote the gene expression and epigenetic modifications mainly addressed to increase neurotrophic factors and neuroprotective functions in the brain, thereby preventing the exacerbation of disease symptoms and course. Among them, the data presented on *ADH1B*, *CYP1A2,* and *MTHFR* depict them as candidate nutrigenomic factors able to impact the susceptibility and progression of NDDs (especially AD and PD). In particular, *ADH1B* is a member of the alcohol dehydrogenase family and is involved in alcohol metabolism. Interestingly, the rs1229984 SNP has been widely investigated in different populations with regard to its functional activity related to the fact that the variant allele of the SNP encodes the most active form of the ADH1B enzyme [99]. In particular, this form may induce a faster biotransformation of alcohol to acetaldehyde. The rs1229984 has been associated with several multifactorial traits and diseases, including cardiovascular disorders, hypertension, migraines, and NDDs [100]. In particular, some studies described this SNP as a susceptibility factor for AD and PD [99,100,101,102]. Indeed, the alternate form of ADH1B may strongly affect acetaldehyde metabolism and accumulation, which may damage the CNS cells and impair cognitive function in the long-term, conferring a higher susceptibility to neurodegenerative and neuroinflammatory conditions responsible for AD and PD onset and progression [61].

Concerning *CYP1A2*, it codes for the cytochrome P450 (CYP1A2) enzyme and it is implicated in the metabolism of caffeine over several drugs [103]. Interestingly, individuals carrying the rs762551 SNP experience a slower caffeine metabolism because of the CYP1A2 decreased activity. On this subject, CYP1A2 is localized in most brain regions and, thus, has been investigated in the context of disease conditions such as AD and PD [103,104,105]. These data highlighted the possible therapeutic potential of caffeine as a neuroprotective factor in AD and PD, as shown by the beneficial effects experienced by patients. Indeed, caffeine compounds have been proposed as a supplement to traditional PD treatment, given its interaction with levodopa and its effect on dyskinesia and gait abnormalities [106]. To this purpose, the investigation of the SNPs of *CYP1A2* could be helpful to provide additional information concerning the possible application of caffeine as supporting treatment for NDDs, taking into account the interindividual variability of patients. On this subject, several studies investigated the association of the rs762551 with PD risk, providing controversial results and raising the need of further research [104,105,106,107,108,109,110]. In fact, The lack of consensus may be due to experimental issues concerning the design of the studies, the homogeneity of the sample cohort, and ethnicity, which appeared to strongly affect the association analysis [111].

The *MTHFR* gene is crucially involved in the processing of amino acids, especially concerning folate metabolism. Genetic variants of the *MTHFR* can affect the proper functioning of this enzyme leading to hyperhomocysteinemia [66]. Interestingly, homocysteine levels have been correlated with structural volume changes in the brain and, therefore, they have been associated with dementia. In addition, temporal lobe atrophy in AD patients has been correlated with hyperhomocysteinemia [112]. Among the most common variants, the rs1801133 has been found to decrease the MTHFR enzyme activity and it has been associated with AD [66,113,114]. In particular, patients with late-onset AD (LOAD) and carrying the *APOE4* risk variant have been found deficient of MTHFR together with decreased levels of S-adenosylmethionine (SAM) in cerebrospinal fluid [66,115]. Given the major role of SAM as a methyl donor within the cells, the hypomethylation resulting from SAM decreasing has been shown to induce the up-regulation of AD-associated genes, leading to Aβ deposition and amyloid angiopathy, as well as tau pathology [115]. Moreover, the rs1801133 has also been associated with PD susceptibility [116,117,118]. Overall, the study of *MTHFR* variants and their functional consequences could be useful to provide a better comprehension of the susceptibility and pathogenic features underlying AD and PD physiopathology, thereby representing additional disease-contributing factors.

Overall, the study of the relationship between *ADH1B*, *CYP1A2*, and *MTHFR* and NDDs, pave the way for further exploring the contribution of nutrigenomics to the susceptibility and progression of NDDs with the purpose of identifying clinically useful biomarkers to be applied in the perspective of the application of precision medicine approaches to patients. Over genetic, epigenetic, and nutrigenomic factors, gut dysbiosis has been associated with many NDDs and several studies suggest that the gut microbiota could represent a stressor target in NDDs. In this regard, the management of dysbiosis represents an additional tool to be further investigated for preventative or treatment purposes. In addition, studies focused on the role of physical activity in neurodegeneration provide evidence for a strong correlation between exercise and NDD; in particular, physical activity acts by directing transcriptional changes in the brain through different pathways across the broad spectrum of neurodegenerative diseases [98]. Physical activity combined with current available therapies, promises to be important for NDDs management and treatment. In conclusion, all of the presented features stand out as potential components to be used for the development of multi-target strategies tailored to provide a more adequate, effective, and personalized treatment to patients suffering from NDDs.

## Data Availability

Not applicable.

## References

[1] Strafella C., Caputo V., Galota M.R., Zampatti S., Marella G., Mauriello S., Cascella R., Giardina E. (2018). Application of Precision Medicine in Neurodegenerative Diseases. Front. Neurol..

[2] Gentile F., Doneddu P.E., Riva N., Nobile-Orazio E., Quattrini A. (2020). Diet, Microbiota and Brain Health: Unraveling the Network Intersecting Metabolism and Neurodegeneration. Int. J. Mol. Sci..

[3] Kamel F. (2013). Paths from Pesticides to Parkinson’s. Science.

[4] Wang M.D., Little J., Gomes J., Cashman N.R., Krewski D. (2017). Identification of risk factors associated with onset and progression of amyotrophic lateral sclerosis using systematic review and meta-analysis. NeuroToxicology.

[5] Gubert C., Kong G., Renoir T., Hannan A.J. (2020). Exercise, diet and stress as modulators of gut microbiota: Implications for neurodegenerative disease. Neurobiol. Dis..

[6] Morris J.K., Bomhoff G.L., Stanford J.A., Geiger P.C. (2010). Neurodegeneration in an animal model of Parkinson’s disease is exacerbated by a high fat diet. Am. J. Phys. Heart Circ. Phys..

[7] Buckman L.B., Hasty A.H., Flaherty D.K., Buckman C.T., Thompson M.M., Matlock B.K., Weller K., Ellacott K.L. (2014). Obesity induced by a high-fat diet is associated with increased immune cell entry into the central nervous system. Brain Behav. Immun..

[8] Hirschberg S., Gisevius B., Duscha A., Haghikia A. (2019). Implications of Diet and The Gut Microbiome in Neuroinflammatory and Neurodegenerative Diseases. Int. J. Mol. Sci..

[9] Scalabrino G. (2009). Vitamin-regulated cytokines and growth factors in the CNS and elsewhere. J. Neurochem..

[10] Ruskin D.N., Svedova J., Cote J.L., Sandau U., Rho J.M., Kawamura M., Boison D., Masino S.A. (2013). Ketogenic diet improves core symptoms of autism in BTBR mice. PLoS ONE.

[11] Beckett T.L., Studzinski C.M., Keller J.N., Murphy M.P., Niedowicz D.M. (2013). A ketogenic diet improves motor performance but does not affect β-amyloid levels in a mouse model of Alzheimer’s disease. Brain Res..

[12] Henderson S.T., Vogel J.L., Barr L.J., Garvin F., Jones J.J., Costantini L.C. (2009). Study of the ketogenic agent AC-1202 in mild to moderate Alzheimer’s disease: A randomized, double-blind, placebo-controlled, multicenter trial. Nutr. Metab..

[13] Genzer Y., Dadon M., Burg C., Chapnik N., Froy O. (2016). Effect of dietary fat and the circadian clock on the expression of brain-derived neurotrophic factor (BDNF). Mol. Cell Endocrinol..

[14] Psaltopoulou T., Sergentanis T.N., Panagiotakos D.B., Sergentanis I.N., Kosti R., Scarmeas N. (2013). Mediterranean diet, stroke, cognitive impairment, and depression: A meta-analysis. Ann. Neurol..

[15] Calon F., Cole G. (2007). Neuroprotective action of omega-3 polyunsaturated fatty acids against neurodegenerative diseases: Evidence from animal studies. Prostaglandins Leukot. Essent. Fat Acids.

[16] Gharekhani A., Khatami M.R., Dashti-Khavidaki S., Razeghi E., Noorbala A.A., Hashemi-Nazari S.S., Mansournia M.A. (2014). The effect of omega-3 fatty acids on depressive symptoms and inflammatory markers in maintenance hemodialysis patients: A randomized, placebo-controlled clinical trial. Eur. J. Clin. Pharmacol..

[17] Gao X., Chen H., Fung T.T., Logroscino G., Schwarzschild M.A., Hu F.B., Ascherio A. (2007). Prospective study of dietary pattern and risk of Parkinson disease. Am. J. Clin. Nutr..

[18] Mattson M.P. (2003). Will caloric restriction and folate protect against AD and PD?. Neurology.

[19] De Crom T.O.E., Mooldijk S.S., Ikram M.K., Ikram M.A., Voortman T. (2022). MIND diet and the risk of dementia: A population-based study. Alzheimers Res. Ther..

[20] Popa-Wagner A., Dumitrascu D.I., Capitanescu B., Petcu E.B., Surugiu R., Fang W.H., Dumbrava D.A. (2020). Dietary habits, lifestyle factors and neurodegenerative diseases. Neural. Regen. Res..

[21] Campbell S.C., Wisniewski P.J., Noji M., McGuinness L.R., Häggblom M.M., Lightfoot S.A., Joseph L.B., Kerkhof L.J. (2016). The effect of diet and exercise on intestinal integrity and microbial diversity in mice. PLoS ONE.

[22] Westfall S., Lomis N., Kahouli I., Dia S.Y., Singh S.P., Prakash S. (2017). Microbiome, probiotics and neurodegenerative diseases: Deciphering the gut brain axis. Cell Mol. Life Sci..

[23] Gareau M.G. (2016). Cognitive function and the microbiome. Int. Rev. Neurobiol..

[24] Bailey M.T., Dowd S.E., Galley J.D., Hufnagle A.R., Allen R.G., Lyte M. (2011). Exposure to a social stressor alters the structure of the intestinal microbiota: Implications for stressor-induced immunomodulation. Brain Behav. Immun..

[25] Bonder M.J., Kurilshikov A., Tigchelaar E.F., Mujagic Z., Imhann F., Vila A.V., Deelen P., Vatanen T., Schirmer M., Smeekens S.P. (2016). The effect of host genetics on the gutmicrobiome. Nat. Genet..

[26] Hu X., Wang T., Jin F. (2016). Alzheimer’s disease and gut microbiota. Sci. China Life Sci..

[27] Wu S.C., Cao Z.S., Chang K.M., Juang J.L. (2017). Intestinal microbial dysbiosis aggravates the progression of Alzheimer’s disease in Drosophila. Nat. Commun..

[28] Terracciano A., Sutin A.R. (2019). Personality and Alzheimer’s disease: An integrative review. Personal. Disord..

[29] DeTure M.A., Dickson D.W. (2019). The neuropathological diagnosis of Alzheimer’s disease. Mol. Neurodegener..

[30] Alzheimer’s association report (2020). 2020 Alzheimer’s disease facts and figures. Alzheimers. Dement..

[31] Jellinger K.A., Janetzky B., Attems J., Kienzl E. (2008). Biomarkers for early diagnosis of Alzheimer disease: ALZheimer ASsociated gene—A new blood biomarker?. J. Cell Mol. Med..

[32] Yacoubian T.A. (2017). Neurodegenerative disorders: Why do we need new therapies?. Drug Discovery Approaches for the Treatment of Neurodegenerative Disorders.

[33] Tudorache I.F., Trusca V.G., Gafencu A.V. (2017). Apolipoprotein E—A Multifunctional Protein with Implications in Various Pathologies as a Result of Its Structural Features. Comput. Struct. Biotechnol. J..

[34] Bruce K.D., Zsombok A., Eckel R.H. (2017). Lipid Processing in the Brain: A Key Regulator of Systemic Metabolism. Front. Endocrinol..

[35] Kim J., Yoon H., Basak J., Kim J. (2014). Apolipoprotein E in synaptic plasticity and Alzheimer’s disease: Potential cellular and molecular mechanisms. Mol. Cells.

[36] Reed B., Villeneuve S., Mack W., DeCarli C., Chui H.C., Jagust W. (2014). Associations between serum cholesterol levels and cerebral amyloidosis. JAMA Neurol..

[37] Estes R.E., Lin B., Khera A., Davis M.Y. (2021). Lipid Metabolism Influence on Neurodegenerative Disease Progression: Is the Vehicle as Important as the Cargo?. Front. Mol. Neurosci..

[38] Moser V.A., Pike C.J. (2017). Obesity Accelerates Alzheimer-Related Pathology in APOE4 but not APOE3 Mice. ENeuro.

[39] Kypreos K.E., Karagiannides I., Fotiadou E.H., Karavia E.A., Brinkmeier M.S., Giakoumi S.M., Tsompanidi E.M. (2009). Mechanisms of obesity and related pathologies: Role of apolipoprotein E in the development of obesity. FEBS J..

[40] Jones N.S., Rebeck G.W. (2018). The Synergistic Effects of APOE Genotype and Obesity on Alzheimer’s Disease Risk. Int. J. Mol. Sci..

[41] Kleinridders A., Ferris H.A., Cai W., Kahn C.R. (2014). Insulin action in brain regulates systemic metabolism and brain function. Diabetes.

[42] Luchsinger J.A., Tang M.X., Shea S., Mayeux R. (2002). Caloric intake and the risk of Alzheimer disease. Arch. Neurol..

[43] Chung K.W., Kim D.H., Park M.H., Choi Y.J., Kim N.D., Lee J., Yu B.P., Chung H.Y. (2013). Recent advances in calorie restriction research on aging. Exp. Gerontol..

[44] Kim S.M., Lim S.M., Yoo J.A., Woo M.J., Cho K.H. (2015). Consumption of high-dose vitamin C (1250 mg per day) enhances functional and structural properties of serum lipoprotein to improve anti-oxidant, anti-atherosclerotic, and anti-aging effects via regulation of anti-inflammatory microRNA. Food Funct..

[45] Monacelli F., Acquarone E., Giannotti C., Borghi R., Nencioni A. (2017). Aging and Alzheimer’s disease. Nutrients.

[46] Vogt N.M., Romano K.A., Darst B.F., Engelman C.D., Johnson S.C., Carlsson C.M., Asthana S., Blennow K., Zetterberg H., Bendlin B.B. (2018). The gut microbiota-derived metabolite trimethylamine N-oxide is elevated in Alzheimer’s disease. Alzheimers Res. Ther..

[47] Liu P., Wu L., Peng G., Han Y., Tang R., Ge J., Zhang L., Jia L., Yue S., Zhou K. (2019). Altered microbiomes distinguish Alzheimer’s disease from amnestic mild cognitive impairment and health in a Chinese cohort. Brain Behav. Immun..

[48] Zhuang Z.Q., Shen L.L., Li W.W., Fu X., Zeng F., Gui L., Lü Y., Cai M., Zhu C., Tan Y.L. (2018). Gut Microbiota is Altered in Patients with Alzheimer’s Disease. J. Alzheimers Dis..

[49] Paley E.L., Merkulova-Rainon T., Faynboym A., Shestopalov V.I., Aksenoff I. (2018). Geographical Distribution and Diversity of Gut Microbial NADH:Ubiquinone Oxidoreductase Sequence Associated with Alzheimer’s Disease. J. Alzheimers Dis..

[50] Vogt N.M., Kerby R.L., Dill-McFarland K.A., Harding S.J., Merluzzi A.P., Johnson S.C., Carlsson C.M., Asthana S., Zetterberg H., Blennow K. (2017). Gut microbiome alterations in Alzheimer’s disease. Sci. Rep..

[51] Parikh I.J., Estus J.L., Zajac D.J., Malik M., Maldonado Weng J., Tai L.M., Chlipala G.E., LaDu M.J., Green S.J., Estus S. (2020). Murine Gut Microbiome Association with APOE Alleles. Front. Immunol..

[52] Chen H., O’Reilly E., McCullough M.L., Rodriguez C., Schwarzschild M.A., Calle E.E., Thun M.J., Ascherio A. (2007). Consumption of dairy products and risk of Parkinson’s disease. Am. J. Epidemiol..

[53] Park M., Ross G.W., Petrovitch H., White L.R., Masaki K.H., Nelson J.S., Tanner C.M., Curb J.D., Blanchette P.L., Abbott R.D. (2005). Consumption of milk and calcium in midlife and the future risk of Parkinson disease. Neurology.

[54] Liu R., Guo X., Park Y., Huang X., Sinha R., Freedman N.D., Hollenbeck A.R., Blair A., Chen H. (2012). Caffeine intake, smoking, and risk of Parkinson disease in men and women. Am. J. Epidemiol..

[55] Ross G.W., Abbott R.D., Petrovitch H., Morens D.M., Grandinetti A., Tung K.-H., Tanner C.M., Masaki K.H., Blanchette P.L., Curb J.D. (2000). Association of Coffee and Caffeine Intake with the Risk of Parkinson Disease. JAMA.

[56] Ascherio A., Zhang S.M., Hernán M.A., Kawachi I., Colditz G.A., Speizer F.E., Willett W.C. (2001). Prospective study of caffeine consumption and risk of Parkinson’s disease in men and women. Ann. Neurol..

[57] Kachroo A., Irizarry M.C., Schwarzschild M.A. (2010). Caffeine protects against combined paraquat and maneb-induced dopaminergic neuron degeneration. Exp. Neurol..

[58] Seidl S.E., Santiago J.A., Bilyk H., Potashkin J.A. (2014). The emerging role of nutrition in Parkinson’s disease. Front Aging Neurosci..

[59] Scheperjans F. (2016). Can microbiota research change our understanding of neurodegenerative diseases?. Neurodegener. Dis. Manag..

[60] Mullins V.A., Bresette W., Johnstone L., Hallmark B., Chilton F.H. (2020). Genomics in Personalized Nutrition: Can You “Eat for Your Genes”?. Nutrients.

[61] Kamal H., Tan G.C., Ibrahim S.F., Shaikh M.F., Mohamed I.N., Mohamed R.M.P., Hamid A.A., Ugusman A., Kumar J. (2020). Alcohol Use Disorder, Neurodegeneration, Alzheimer’s and Parkinson’s Disease: Interplay Between Oxidative Stress, Neuroimmune Response and Excitotoxicity. Front. Cell Neurosc..

[62] Sarparast M., Dattmore D., Alan J., Lee K.S.S. (2020). Cytochrome P450 Metabolism of Polyunsaturated Fatty Acids and Neurodegeneration. Nutrients.

[63] Cajavilca C.E., Gadhia R.R., Román G.C. (2019). MTHFR Gene Mutations Correlate with White Matter Disease Burden and Predict Cerebrovascular Disease and Dementia. Brain Sci..

[64] Caputo V., Strafella C., Termine A., Fabrizio C., Ruffo P., Cusumano A., Giardina E., Ricci F., Cascella R. (2021). Epigenomic signatures in age-related macular degeneration: Focus on their role as disease modifiers and therapeutic targets. Eur. J. Ophthalmol..

[65] Krautkramer K.A., Kreznar J.H., Romano K.A., Vivas E.I., Barrett-Wilt G.A., Rabaglia M.E., Keller M.P., Attie A.D., Rey F.E., Denu J.M. (2016). Diet-Microbiota Interactions Mediate Global Epigenetic Programming in Multiple Host Tissues. Mol. Cell.

[66] Grazioli E., Dimauro I., Mercatelli N., Wang G., Pitsiladis Y., Di Luigi L., Caporossi D. (2017). Physical activity in the prevention of human diseases: Role of epigenetic modifications. BMC Genom..

[67] Zimmer P., Baumann F.T., Bloch W., Schenk A., Koliamitra C., Jensen P., Mierau A., Hülsdünker T., Reinart N., Hallek M. (2014). Impact of exercise on pro inflammatory cytokine levels and epigenetic modulations of tumor-competitive lymphocytes in non-Hodgkin-lymphoma patients-randomized controlled trial. Eur. J. Haematol..

[68] Lavratti C., Dorneles G., Pochmann D., Peres A., Bard A., de Lima Schipper L., Dal Lago P., Wagner L.C., Elsner V.R. (2017). Exercise-induced modulation of histone H4 acetylation status and cytokines levels in patients with schizophrenia. Physiol. Behav..

[69] Yao B., Christian K.M., He C., Jin P., Ming G.L., Song H. (2016). Epigenetic mechanisms in neurogenesis. Nat. Rev. Neurosci..

[70] Matsumoto L., Takuma H., Tamaoka A., Kurisaki H., Date H., Tsuji S., Iwata A. (2010). CpG demethylation enhances alpha-synuclein expression and affects the pathogenesis of Parkinson’s disease. PLoS ONE.

[71] International Parkinson’s Disease Genomics Consortium (IPDGC), Wellcome Trust Case Control Consortium 2 (WTCCC2) (2011). A two-stage meta-analysis identifies several new loci for Parkinson’s disease. PLoS Genet..

[72] Eryilmaz I.E., Cecener G., Erer S., Egeli U., Tunca B., Zarifoglu M., Elibol B., Tokcaer B.A., Saka E., Demirkiran M. (2017). Epigenetic approach to early-onset Parkinson’s disease: Low methylation status of SNCA and PARK2 promoter regions. Neurol. Res..

[73] Coppedè F. (2012). Genetics and epigenetics of Parkinson’s disease. Sci. World J..

[74] Chen X., Xie C., Tian W., Sun L., Zheng W., Hawes S., Chang L., Kung J., Ding J., Chen S. (2020). Correction to: Parkinson’s disease-related Leucine-rich repeat kinase 2 modulates nuclear morphology and genomic stability in striatal projection neurons during aging. Mol. Neurodegener..

[75] Goldstein O., Gana-Weisz M., Casey F., Meltzer-Fridrich H., Yaacov O., Waldman Y.Y., Lin D., Mordechai Y., Zhu J., Cullen P.F. (2021). PARK16 locus: Differential effects of the non-coding rs823114 on Parkinson’s disease risk, RNA expression, and DNA methylation. J. Genet. Genomics.

[76] Brini S., Sohrabi H.R., Peiffer J.J., Karrasch M., Hämäläinen H., Martins R.N., Fairchild T.J. (2018). Physical activity in preventing Alzheimer’s disease and cognitive decline: A narrative review. Sports Med..

[77] Crowley E.K., Nolan Y.M., Sullivan A.M. (2018). Neuroprotective effects of voluntary running on cognitive dysfunction in an α-synuclein rat model of Parkinson’s disease. Neurobiol. Aging.

[78] Fritz N.E., Rao A.K., Kegelmeyer D., Kloos A., Busse M., Hartel L., Carrier J., Quinn L. (2017). Physical therapy and exercise interventions in huntington’s disease: A mixed methods systematic review. J. Huntingt. Dis..

[79] Cass S.P. (2017). Alzheimer’s disease and exercise: A literature review. Curr. Sports Med. Rep..

[80] Hoffman-Goetz L., Pervaiz N., Packer N., Guan J. (2010). Freewheel training decreases pro- and increases anti-inflammatory cytokine expression in mouse intestinal lymphocytes. Brain Behav. Immun..

[81] Packer N., Hoffman-Goetz L. (2012). Apoptotic and inflammatory cytokine protein expression in intestinal lymphocytes after acute treadmill exercise in young and old mice. J. Sports Med. Phys. Fit..

[82] Lambert C.P., Wright N.R., Finck B.N., Villareal D.T. (2008). Exercise but not diet-induced weight loss decreases skeletal muscle inflammatory gene expression in frail obese elderly persons. J. Appl. Physiol..

[83] Clark A., Mach N. (2016). Exercise-induced stress behavior, gut-microbiota-brain axis and diet: A systematic review for athletes. J. Int. Soc. Sports Nutr..

[84] Colovati M., Novais I.P., Zampol M., Mendes G.D., Cernach M., Zanesco A. (2020). Interaction between physical exercise and APOE gene polymorphism on cognitive function in older people. Braz. J. Med. Biol. Res..

[85] Diniz T.G., Silva A.S., Dos Santos Nunes M.K., Ribeiro M.D., Filho J.M., do Nascimento R.A.F., Gomes C.N.A.P., Evangelista I.W.Q., de Oliveira N.F.P., Persuhn D.C. (2021). Physical Activity Level Influences *MTHFR* Gene Methylation Profile in Diabetic Patients. Front. Physiol..

[86] Deminice R., Ribeiro D.F., Frajacomo F.T. (2016). The Effects of Acute Exercise and Exercise Training on Plasma Homocysteine: A Meta-Analysis. PLoS ONE.

[87] Erruzzi I., Senesi P., Montesano A., La Torre A., Alberti G., Benedini S., Caumo A., Fermo I., Luzi L. (2011). Genetic polymorphisms of the enzymes involved in DNA methylation and synthesis in elite athletes. Physiol. Genom..

[88] Zarebska A., Ahmetov I.I., Sawczyn S., Weiner A.S., Kaczmarczyk M., Ficek K., Maciejewska-Karlowska A., Sawczuk M., Leonska-Duniec A., Klocek T. (2014). Association of the MTHFR 1298A>C (rs1801131) polymorphism with speed and strength sports in Russian and Polish athletes. J. Sports Sci..

[89] Dinç N., Yücel S.B., Taneli F., Sayın M.V. (2016). The effect of the MTHFR C677T mutation on athletic performance and the homocysteine level of soccer players and sedentary individuals. J. Hum. Kinet..

[90] Dankner R., Chetrit A., Dror G.K., Sela B.A. (2007). Physical activity is inversely associated with total homocysteine levels, independent of C677T MTHFR genotype and plasma B vitamins. Age.

[91] Barreto G., Grecco B., Merola P., Reis C.E.G., Gualano B., Saunders B. (2021). Novel insights on caffeine supplementation, CYP1A2 genotype, physiological responses and exercise performance. Eur. J. Appl. Physiol..

[92] Guest N., Corey P., Vescovi J., El-Sohemy A. (2018). Caffeine, CYP1A2 Genotype, and Endurance Performance in Athletes. Med. Sci. Sports Exerc..

[93] Soares R.N., Schneider A., Valle S.C., Schenkel P.C. (2018). The influence of CYP1A2 genotype in the blood pressure response to caffeine ingestion is affected by physical activity status and caffeine consumption level. Vasc. Pharm..

[94] Grgic J., Pickering C., Del Coso J., Schoenfeld B.J., Mikulic P. (2021). CYP1A2 genotype and acute ergogenic effects of caffeine intake on exercise performance: A systematic review. Eur. J. Nutr..

[95] Lin E., Kuo P.H., Liu Y.L., Yang A.C., Tsai S.J. (2019). Association and Interaction Effects of Interleukin-12 Related Genes and Physical Activity on Cognitive Aging in Old Adults in the Taiwanese Population. Front. Neurol..

[96] Griffin W.S. (2013). Neuroinflammatory cytokine signaling and Alzheimer’s disease. N. Engl. J. Med..

[97] Papenberg G., Ferencz B., Mangialasche F., Mecocci P., Cecchetti R., Kalpouzos G., Fratiglioni L., Bäckman L. (2016). Physical activity and inflammation: Effects on gray-matter volume and cognitive decline in aging. Hum. Brain Mapp..

[98] Santiago J.A., Quinn J.P., Potashkin J.A. (2022). Physical Activity Rewires the Human Brain against Neurodegeneration. Int. J. Mol. Sci..

[99] García-Martín E., Diez-Fairen M., Pastor P., Gómez-Tabales J., Alonso-Navarro H., Alvarez I., Cárcel M., Aguilar M., Agúndez J.A., Jiménez-Jiménez F.J. (2019). Association between the missense alcohol dehydrogenase rs1229984T variant with the risk for Parkinson’s disease in women. J. Neurol..

[100] Ma L., Lu Z.N. (2016). Role of *ADH1B rs1229984* and *ALDH2 rs671* gene polymorphisms in the development of Alzheimer’s disease. Genet. Mol. Res..

[101] Jin X., Long T., Chen H., Zeng Y., Zhang X., Yan L., Wu C. (2022). Associations of Alcohol Dehydrogenase and Aldehyde Dehydrogenase Polymorphism with Cognitive Impairment Among the Oldest-Old in China. Front. Aging Neurosci..

[102] Almeida O.P., Hankey G.J., Yeap B.B., Golledge J., Flicker L. (2014). Alcohol consumption and cognitive impairment in older men: A mendelian randomization study. Neurology.

[103] Kolahdouzan M., Hamadeh M.J. (2017). The neuroprotective effects of caffeine in neurodegenerative diseases. CNS Neurosci. Ther..

[104] Kim I.Y., O’Reilly É.J., Hughes K.C., Gao X., Schwarzschild M.A., McCullough M.L., Hannan M.T., Betensky R.A., Ascherio A. (2018). Interaction between caffeine and polymorphisms of glutamate ionotropic receptor NMDA type subunit 2A (GRIN2A) and cytochrome P450 1A2 (CYP1A2) on Parkinson’s disease risk. Mov. Disord..

[105] Siokas V., Aloizou A.M., Tsouris Z., Liampas I., Liakos P., Calina D., Docea A.O., Tsatsakis A., Bogdanos D.P., Hadjigeorgiou G.M. (2021). ADORA2A rs5760423 and CYP1A2 rs762551 Polymorphisms as Risk Factors for Parkinson’s Disease. J. Clin. Med..

[106] Negida A., Elfil M., Attia A., Farahat E., Gabr M., Essam A., Attia D., Ahmed H. (2017). Caffeine; the Forgotten Potential for Parkinson’s Disease. CNS Neurol. Disord. Drug Targets.

[107] Palacios N., Weisskopf M., Simon K., Gao X., Schwarzschild M., Ascherio A. (2010). Polymorphisms of caffeine metabolism and estrogen receptor genes and risk of Parkinson’s disease in men and women. Parkinsonism Relat. Disord..

[108] Popat R.A., Van Den Eeden S.K., Tanner C.M., Kamel F., Umbach D.M., Marder K., Mayeux R., Ritz B., Ross G.W., Petrovitch H. (2011). Coffee, ADORA2A, and CYP1A2: The caffeine connection in Parkinson’s disease. Eur. J. Neurol..

[109] Chuang Y.H., Lill C.M., Lee P.C., Hansen J., Lassen C.F., Bertram L., Greene N., Sinsheimer J.S., Ritz B. (2016). Gene-Environment Interaction in Parkinson’s Disease: Coffee, ADORA2A, and CYP1A2. Neuroepidemiology.

[110] Hill-Burns E.M., Hamza T.H., Zabetian C.P., Factor S.A., Payami H. (2011). An attempt to replicate interaction between coffee and *CYP1A2* gene in connection to Parkinson’s disease. Eur. J. Neurol..

[111] Denden S., Bouden B., Haj Khelil A., Ben Chibani J., Hamdaoui M.H. (2016). Gender and ethnicity modify the association between the CYP1A2 rs762551 polymorphism and habitual coffee intake: Evidence from a meta-analysis. Genet. Mol. Res..

[112] You M., Zhou X., Yin W., Wan K., Zhang W., Li C., Li M., Zhu W., Zhu X., Sun Z. (2021). The Influence of MTHFR Polymorphism on Gray Matter Volume in Patients with Amnestic Mild Cognitive Impairment. Front. Neurosci..

[113] Belcavello L., Camporez D., Almeida L.D., Morelato R.L., Batitucci M.C., de Paula F. (2015). Association of MTHFR and PICALM polymorphisms with Alzheimer’s disease. Mol. Biol. Rep..

[114] Stoccoro A., Tannorella P., Salluzzo M.G., Ferri R., Romano C., Nacmias B., Siciliano G., Migliore L., Coppedè F. (2017). The Methylenetetrahydrofolate Reductase C677T Polymorphism and Risk for Late-Onset Alzheimer’s disease: Further Evidence in an Italian Multicenter Study. J. Alzheimers Dis..

[115] Zhuo J.M., Praticò D. (2010). Acceleration of brain amyloidosis in an Alzheimer’s disease mouse model by a folate, vitamin B6 and B12-deficient diet. Exp. Gerontol..

[116] Romero-Gutiérrez E., Vázquez-Cárdenas P., Moreno-Macías H., Salas-Pacheco J., Tusié-Luna T., Arias-Carrión O. (2021). Differences in MTHFR and LRRK2 variant’s association with sporadic Parkinson’s disease in Mexican Mestizos correlated to Native American ancestry. NPJ Parkinsons Dis..

[117] Zhu Z.G., Ai Q.L., Wang W.M., Xiao Z.C. (2013). Meta-analysis supports association of a functional SNP (rs1801133) in the MTHFR gene with Parkinson’s disease. Gene.

[118] Liu L., Zhang L., Guo L., Yu Q., Li H., Teng J., Xie A. (2018). MTHFR C677T and A1298C polymorphisms may contribute to the risk of Parkinson’s disease: A meta-analysis of 19 studies. Neurosci. Lett..

